# MicroRNA-34a and microRNA-21 play roles in the chemopreventive effects of 3,6-dihydroxyflavone on 1-methyl-1-nitrosourea-induced breast carcinogenesis

**DOI:** 10.1186/bcr3194

**Published:** 2012-05-22

**Authors:** Chang Hui, Fu Yujie, Yuan Lijia, Yi Long, Xu Hongxia, Zhou Yong, Zhu Jundong, Zhang Qianyong, Mi Mantian

**Affiliations:** 1 Chongqing Key Laboratory of Nutrition and Food Safety, Research Center for Nutrition and Food Safety, Third Military Medical University, 30 Gaotanyan Street, Shapingba District, Chongqing 400038, China

## Abstract

**Introduction:**

miRNAs are very important regulators in biological processes such as development, cellular differentiation, and carcinogenesis. Given the important role of miRNAs in tumorigenesis and development, it is worth investigating whether some miRNAs play roles in the anticancer mechanism of flavonoids. However, such a role has not yet been reported. We previously selected the promising anticancer agent 3,6-dihydroxyflavone (3,6-DHF) in pharmacodynamic experiments, which may serve as a leading compound for developing more potent anticancer drugs or chemopreventive supplements. The present study aims to investigate the chemopreventive activities of 3,6-DHF against mammary carcinogenesis.

**Methods:**

The experimental model of breast carcinogenesis was developed by intraperitoneal injection of 1-methyl-1-nitrosourea (MNU). The bioavailability of 3,6-DHF in rats was detected by HPLC. The expression of microRNA-34a (miR-34a) and microRNA-21 (miR-21) was evaluated by real-time quantitative RT-PCR. Cell apoptosis was analyzed by flow cytometry or terminal deoxynucleotidyl transferase dUTP nick end-labeling assay. The mitochondrial membrane potential was assayed using 5,5',6,6'-tetrachloro-1,1',3,3'-tetraethyl-imidacarbocyanine iodide dye by confocal laser scanning microscopy. The level of cytochrome C in cytosol was evaluated by western blotting.

**Results:**

Our study showed that oral administration of 3,6-DHF effectively suppressed MNU-induced breast carcinogenesis in rats, decreasing the cancer incidence by 35.7%. The detection of bioavailability indicated that the concentration of 3,6-DHF was 2.5 ± 0.4 μg/ml in plasma of rats within 2 hours after administration, and was 21.7 ± 3.8 μg/ml in urine within 24 hours. Oral administration of 3,6-DHF to BALB/c nude mice bearing breast cancer cell xenografts also significantly suppressed tumor growth *in vivo*. Furthermore, our study revealed that the global upregulation of miR-21 and downregulation of miR-34a in breast carcinogenesis could be reversed by 3,6-DHF, which significantly upregulated miR-34a expression and decreased miR-21 expression - inducing apoptosis of breast cancer cells *in vitro *and *in vivo*. Overexpression of miR-34a induced by plasmid transfection or inhibition of miR-21 by oligonucleotides markedly promoted the pro-apoptotic effect of 3,6-DHF. Inactivation of miR-34a or overproduction of miR-21 compromised the anticancer effects of 3,6-DHF.

**Conclusion:**

These findings indicate that 3,6-DHF is a potent natural chemopreventive agent, and that miR-34a and miR-21 play roles in MNU-induced breast carcinogenesis and the anticancer mechanism of flavonoids.

## Introduction

Breast cancer is the leading cause of cancer death in women worldwide due to the complicated etiology involving both genetic and environmental factors. About 70,000 new cases of breast cancer are likely to be diagnosed annually. Understanding the signaling pathways involved in breast carcinogenesis is important for the development of more effective tumor prevention and therapies. miRNAs are a class of noncoding RNAs 18 to 25 nucleotides long that negatively regulate gene expression by binding to the 3'-UTR of target messenger mRNAs, causing translational repression or degradation. Deregulation of miRNAs has been demonstrated to play roles in the pathogenesis of human diseases, including malignancy [1,2]. Some miRNAs have been identified from cancers and appear to play crucial roles in proliferation, differentiation, and apoptosis [3-5]. One of these, microRNA-21 (miR-21), is a key player. As an anti-apoptosis factor, aberrantly expressed miR-21 compromises tumor suppressor-mediated apoptosis of cancer cells [6,7]. However, more studies are needed to define the functional role of miR-21 in breast tumorigenesis.

p53 is a potent tumor suppressor. Activated p53 elicits a plethora of biochemical and biological outcomes, ranging from repair of minor damage to cessation of cell-cycle progression, induction of replicative senescence, and apoptosis. The p53 inactivation is one of the most frequent genetic alterations in human cancer [8,9]. Studies have reported that, in addition to regulating the expression of hundreds of protein-coding genes, p53 also modulates the levels of miRNAs. Among those p53-related miRNAs, microRNA-34a (miR-34a) is a typical one. As a pro-apoptotic transcriptional target of p53, miR-34a exerts anti-proliferative effects and contributes to p53-mediated apoptosis [10,11]. Monitoring miR-34a expression in human tumors and manipulating its levels may provide new clues for cancer prevention, diagnosis, and therapy. Given the importance of pro-apoptosis activity of miR-34a and anti-apoptosis activity of miR-21 in cancer cells, it is interesting to investigate their roles in carcinogenesis. We hypothesize that the disbalance of cell apoptosis signaling regulation plays an important role in breast tumorigenesis.

Flavonoids, a class of natural polyphenolic compounds, are widely found in abundance in fruits, vegetables, and other vegetable diets. Epidemiological investigation and experimental studies have confirmed that plant flavonoids have extensive biological benefits such as antioxidant, anti-inflammatory, anti-tumor, and anti-atherosclerotic properties [12-15]. Recent recognition of the fact that a diet rich in plant foods lowers the risks of cancer arouses interest in isolating and characterizing the nutritive and non-nutritive components in fruits, vegetables, and cereals for potential chemopreventive and pharmaceutical agents. Development of anticancer agents from flavonoids and other natural products has currently become a very hot topic. Our preliminary screening of anticancer drugs from 23 flavonoid compounds identified 3,6-dihydroxyflavone (3,6-DHF) as a promising anticancer agent with a very strong cytotoxicity against breast cancer cells, which may serve as a leading compound for developing more potent anticancer drugs or chemopreventive supplements [16,17]. However, whether 3,6-DHF possesses effective anticancer properties *in vivo *and its chemopreventive activities against mammary carcinogenesis remain unclear. Furthermore, given the important role of miRNAs in tumorigenesis and development, it is worth investigating whether some miRNAs play roles in the anticancer mechanism of flavonoids. However, such a role has not yet been reported.

In this study, we evaluated the chemopreventive effect of 3,6-DHF on mammary carcinogenesis, and investigated the roles of miR-34a and miR-21 in the anticancer mechanism of flavonoid.

## Materials and methods

### Animals

Female Sprague-Dawley rats (age 42 to 48 days, weight 145 to 165 g) and BALB/c nude mice (age 42 to 48 days, weight 15 to 20 g) were obtained from the Medical Experimental Animal Center of the Third Military Medical University (SCXK-(army)-2007-015). The rats were bred and maintained in accordance with our institutional guidelines for the use of laboratory animals. Animal rooms were maintained at 25°C with 50% relative humidity and a 12-hour light/12-hour dark cycle. All animal procedures were approved by the Animal Ethics Committee of the Third Military Medical University.

### Cell lines

Human breast cancer cell lines MDA-MB-453 and MDA-MB-231 were purchased from the Institute of Biochemistry and Cell Biology, Chinese Academy of Sciences (Shanghai, China). MDA-MB-453 cells were grown in RPMI 1640 medium and MDA-MB-231 cells were grown in DMEM/F12 medium supplemented with 10% fetal bovine serum in a humidified atmosphere of 5% CO_2_/95% air at 37°C. The human vascular endothelial cell line EA.hy926 was grown in DMEM medium supplemented with 100 U/ml penicillin, 100 μg/ml streptomycin and 10% fetal bovine serum at 37°C under a humidified atmosphere with 5% CO_2_/95% air.

### Chemicals and reagents

Growth media supplemented with 2 mM glutamine and fetal bovine serum were purchased from Hyclone (Beijing, China). The Annexin V-FITC Vybrant Apoptosis Assay Kit and the 5,5',6,6'-tetrachloro-1,1',3,3'-tetraethyl-imidacarbocyanine iodide (JC-1) dye were purchased from Molecular Probes Inc. (Eugene, OR, USA). Antibodies of cytochrome C were purchased from Cell Signaling Technology Inc. (Danvers, MA, USA). The *in situ *cell death detection kit (terminal deoxynucleotidyl transferase-mediated dUTP nick end-labeling (TUNEL)) was purchased from Roche Diagnostics (Mannheim, Germany). Trizol reagent, lipofectamine 2000 and Opti-Mem were purchased from Invitrogen (Carlsbad, CA, USA). 3,6-DHF, 1-methyl-1-nitrosourea (MNU), 3-(4,5-dimethylthiazol-2-yl)-2,5-diphenyl tetrazolium bromide (MTT), dimethylsulfoxide, PBS and other chemicals were purchased from Sigma-Aldrich (St. Louis, MO, USA).

### Experimental model of carcinogenesis

The rats were acclimatized on an AIN-93G diet for 3 days and randomly divided into four groups: normal, control, low-dosage treatment and high-dosage treatment groups. Rats in the low-dosage and the high-dosage treatment groups were fed intragastrically with 10 and 20 mg/kg/day 3,6-DHF, respectively; rats in the normal and the control groups were fed orally (intragastrically) with vehicle alone (normal saline). One week later, except for the normal group, all rats were given a single intraperitoneal injection of MNU (50 mg/kg body weight) that was dissolved in physiological saline (10 mg/ml) containing 0.05% acetic acid and used within 30 minutes after preparation. Rats in the normal group were injected with an equal volume of physiological saline. During the experiments, the rats were weighed twice per week and palpated once per day. The rats were monitored for mammary tumor development throughout the study and at sacrifice. The following parameters were calculated: tumor latency, the average time of tumor appearance; tumor incidence, the percentage of rats bearing at least one palpable malignant mammary tumor; tumor multiplicity, the total number of malignant mammary tumors in each group; and tumor volume and weight, the average volume and weight of all malignant tumors in each experimental group.

### Detection of miR-34a and miR-21 expression in breast tumors

A total of 30 rats were randomly divided into control and treatment groups. Animals were treated in the way described above for experimental models of tumorigenesis. Rats in the treatment group were fed with 20 mg/kg/day 3,6-DHF. After MNU injection for 0, 4, 8 and 18 weeks, three rats in each group were randomly selected out respectively and were sacrificed. Breast tissue (at 0, 4 and 8 weeks) and tumors (at 18 weeks) were clipped for total RNA extraction. The expression of miR-34a and miR-21 was detected by quantitative RT-PCR as mentioned below.

### HPLC analysis of 3,6-dihydroxyflavone

The urine sample or plasma sample (1.0 ml) was added with 5 μl β-glucuronidase (≥2,000 units/ml), 5 μl sulfatase (≥8,500 units/ml) and 1.5 ml sodium acetate solution (0.2 M), and then incubated in water for 30 minutes at 37°C. The mixed solution was added with 5.0 ml ethyl acetate (0.2 M), and centrifuged (3,000 rpm) for 10 minutes at 4°C. The supernatant solution was dried under reduced pressure for 5 hours at 40°C. The residue was dissolved with methanol and treated with 0.45 μm membrane filtration, and then analyzed by HPLC. HPLC analyses were carried out on a Waters 1525 series liquid chromatography system equipped with a dual λ absorbance detector (Waters, Milford, MA, USA). The analyses were performed with a Hyper 0DS2 C18 column (4.6 mm × 50 mm, 1.8 μm; Waters, Milford, MA, USA) with 0.5% ethanoic acid or methanol as the mobile phase. Elution started at 15% (methanol), increasing to 67% within 40 minutes at a flow rate of 1.0 ml/minute. 3,6-DHF was quantified based on the peak area of the respective standard curves measured at 270 nm. Typically, 20 μl was injected for the analyses.

### Anti-tumor effect of 3,6-dihydroxyflavone on xenografted MDA-MB-453 cells in athymic mice

Female immunodeficient BALB/c nude mice were randomized and then implanted with MDA-MB-453 cells at a density of 2 × 10^6 ^cells/ml subcutaneously into the right axilla. Mice were fed orally (intragastrically) 72 hours later with 3,6-DHF (10 or 20 mg/kg/day; *n *= 6) or vehicle alone (normal saline; *n *= 6). Tumor size was measured once every 4 days in two perpendicular dimensions with vernier calipers and converted to tumor volume (*V*_t_) using the formula: (L×W^2^)/2, where L and W refer to the longer and shorter dimensions, respectively.

The results are expressed as the ratio of median tumor volumes of treated animals and of control animals (*T*/*C*):

$$T/C\left(\%\right)\;=\text{ median }V_{\text{t}}\text{ of treated animals }/\text{ median }V_{\text{t}}\text{ of control animals}\times \text{1}00$$

### Cell viability assay

Cell viability was assessed by MTT assay as described previously [18]. Cells in the logarithmic growth phase were plated in 24-well plates at a density of 10^5 ^cells/well. At the end of the treatment, 40 μl MTT (5 mg/ml) were added to each well and the cells were cultured for another 4 hours. The formazan crystals were dissolved in dimethylsulfoxide, and the absorbance of each well was measured at 490 nm on a Bio-Rad automatic EIA analyzer (Bio-Rad, Hercules, CA, USA).

### Detection of apoptosis by flow cytometry

Treated cells were washed three times with ice-cold PBS, and stained with Annexin V-FITC for 15 minutes at room temperature in 200 μl binding buffer. An additional 300 μl binding buffer was then added. The cells were stained with propidium iodide for 30 minutes at 4°C and the fluorescence intensity was determined by flow cytometry.

### Detection of apoptosis by TUNEL assay

Apoptosis was determined using the TUNEL assay for the identification of double-stranded DNA breaks according to the manufacturer's instructions. Briefly, tissue paraffin sections were washed three times with PBS for 10 minutes each time, dehydrated for 2 minutes in absolute ethanol and then treated with permeabilization solution (1% Triton X-100 in 1% sodium citrate) for 15 minutes at room temperature. Strand breaks were labeled with fluoresceinated dUTP and visualized following reaction with antifluorescein antibody conjugated with horseradish peroxidase and diaminobenzidine substrate. All slides were counter-stained using hematoxylin.

### Mitochondrial membrane potential assay

Treated cells were washed twice and incubated in complete medium containing 10 μg fluorescent lipophilic cationic JC-1 dye for 30 minutes at 37°C in the dark. Stained cells were harvested, washed, resuspended, and subjected to immediate analysis by a laser confocal scanning microscopy. JC-1 was selectively accumulated in intact mitochondria and formed multimer J-aggregates that fluoresced red (590 nm) at a high membrane potential. Monomeric JC-1 fluoresced green (527 nm) at a low membrane potential. The different colors for fluorescence of JC-1 thus represented different mitochondrial membrane potentials.

### Western blot analysis

Treated cells were lysed in cytosol-extracting buffer (10 mM HEPES, 10 mM KCl, 0.1 mM ethylenediamine tetracetic acid, 1.5 mM MgCl_2_, 0.2% NP40, 1 mM dithiothreitol, and 0.5 mM phenylmethylsulfonyl fluoride) for 2 minutes, and supernatants (cytosolic fraction) were collected by centrifugation at 12,000 rpm for 5 minutes at 4°C [19]. Cytosolic extracts were resolved in a 12.5% polyacrylamide gel and transferred, and the blot was hybridized with cytochrome C antibody. The signal was developed using an ECL kit and the relative photographic density was quantified by LAS-3000 Image Reader (Fujifilm, Tokyo, Japan).

### Plasmids, oligonucleotides and transfections

To construct plasmids expressing miR-34a and miR-21, the genomic fragment containing miR-34a and miR-21 precursor was amplified from MDA-MB-453 cells and cloned into the pcDNA6.2-GW/EmGFP vector. The PCR primers are as follows: miR-34a, forward 5'-TTTAAGCTTATGCGCCCTGCC-3' and reverse 5'-TTTCTCGAGAGAGCTTCCGAAGTCCTGG-3'; and miR-21, forward 5'-GAATTCCGATCTTAACAGGCCAGAAATGC-3' and reverse 5'-AGATCTCCACCAGACAGAAGGACCAGAGT-3'. The clones were confirmed by restriction analysis and DNA sequencing of both strands (Invitrogen, Shanghai, China). Anti-miR-34a oligonucleotides (5'-ACAACCAGCTAAGACACTGCC-3') were from Exiqon (Vedbaek, Denmark). 2'-*O*-methyl-anti-miR-21 (5'-UCAUACAGCUAGAUAACCAAAGA-3') was chemically synthesized by Genechem (Shanghai, China). Cells were transfected using Lipofectamine 2000 in Opti-Mem (Invitrogen, Carlsbad, CA, USA), according to the manufacturer's protocol. The medium was replaced 8 hours later and cells were collected for the next experiments 48 hours post-transfection. The final concentrations of oligonucleotides were 100 nM.

### Quantitative RT-PCR analysis of miR-34a and miR-21 expression

Total RNA was extracted using Trizol reagent. For detection of miR-34a and miR-21 expression, stem-loop RT-PCR was performed as previously described [20,21]. Quantitative PCR was carried out using a BioEasy SYBR green I real-time PCR kit (Bioer, Hangzhou, China) according to the manufacturer's protocol. Relative expression was evaluated by comparative computed tomography method and was normalized to the expression of U6 small RNA. The primers for miR-34a are: stem-loop RT primer 5'-GTCGTATCCAGTGCAGGGTCCGAGGTATTCGCACTGGATACGACAACAAC-3', forward 5'-CGGTATCATTTGGCAGTGTCT-3' and reverse 5'-GTGCAGGGTCCGAGGT-3'. The primers for miR-21 are: RT primer 5'-GTCGTATCCAGTGCAGGGTCCGAGGTATTCGCACTGGATACGACTCAACA-3', forward 5'-GCCCGCTAGCTTATCAGACTGATG-3' and reverse 5'-GTGCAGGGTCCGAGGT-3'. The primers for U6 are: RT primer 5'-GTCGTATCCAGTGCAGGGTCCGAGGTATTCGCACTGGATACGACAAAAATATG-3', forward 5'-GCGCGTCGTGAAGCGTTC-3' and reverse 5'-GTGCAGGGTCCGAGGT-3'. All quantitative RT-PCRs were performed in triplicate.

### Statistical analysis

Statistical analyses were performed using the SPSS 11.0 package (SPSS Inc., Chicago, IL, USA). Results are presented as the mean ± standard deviation, for at least three independent experiments. Tumor incidences were compared using the chi-squared test. Other data were analyzed by one-way analysis of variance followed by Tukey's test for multiple comparisons. Significance of difference was set at *P *< 0.05.

## Results

### Bioavailability of 3,6-dihydroxyflavone in rats

**Figure 1:**
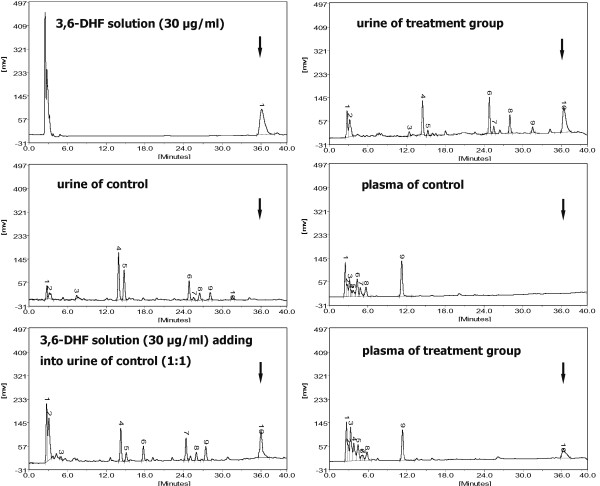
**Spectrum of graphs for HPLC analysis**. After administration of 3,6-dihydroxyflavone (3,6-DHF) (20 mg/kg/day), plasma within 2 hours and urine within 24 hours of the rats were collected and analyzed by HPLC (*n *= 6). Arrows indicate peaks of 3,6-DHF.

After administration of 3,6-DHF (20 mg/kg/day), the plasma and urine of the rats were collected and analyzed by HPLC (Figure 1). The concentration of 3,6-DHF in the plasma within 2 hours after ingestion was 2.5 ± 0.4 μg/ml, and the average concentration of 3,6-DHF in the urine within 24 hours was 21.7 ± 3.8 μg/ml.

### Chemopreventive effects of 3,6-dihydroxyflavone against breast carcinogenesis in rats

Three end points of the carcinogenic response in MNU-treated groups were measured: tumor incidence (number of tumor-bearing rats divided by the total number of rats in a group), tumor latency (time to detection of a palpable mammary tumor), and tumor multiplicity (average number of tumors per rat). As shown in Table 1, the tumor incidence and multiplicity in the high-dosage group were significantly lower than in the control group. The cancer incidence was decreased by 35.7%, indicating that oral administration of 3,6-DHF (20 mg/kg/day) can effectively suppress MUN-induced mammal carcinogenesis. No evidence of adverse effects was observed in rats administered with 3,6-DHF orally and they grew at the same rate as the controls.

**Table 1:** Effect of 3,6-dihydroxyflavone on cancer endpoints and body weight

Group	Total number of rats	Number of rats with tumor	Incidence (%)	Multiplicity (average number/rat)	Latency (days)	Final body weight (g)
No treatment	8	0	0	-	-	291.3 ± 5.2
MNU only	14	13	92.8	2.6 ± 0.6	74.4 ± 7.1	281.7 ± 4.8
MNU + 10 mg/kg 3,6-DHF	14	9	64.3	2.2 ± 0.3	80.8 ± 7.8	275.5 ± 5.8
MNU + 20 mg/kg 3,6-DHF	14	8*	57.1*	1.9 ± 0.4*	87.5 ± 5.1**	278.7 ± 5.1

Data presented as mean ± standard deviation. *P < 0.05, **P < 0.01 compared with the 1-methyl-1-nitrosourea (MNU)-only treatment group. Tumor incidence in the MNU + 20 mg/kg 3,6-dihydroxyflavone (3,6-DHF) treatment group was significantly lower than that in the MNU-only treatment group, indicating that oral administration of 3,6-DHF (20 mg/kg/day) can effectively suppress MUN-induced mammal carcinogenesis.

### 3,6-Dihydroxyflavone inhibits the growth of breast cancer cells *in vivo*

**Figure 2:**
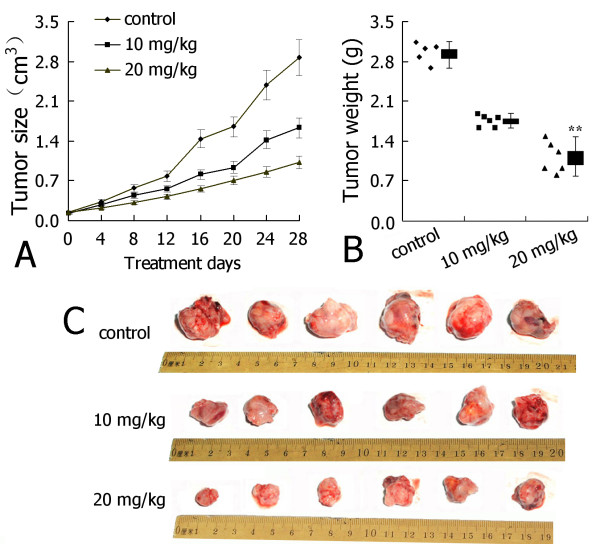
**3,6-Dihydroxyflavone significantly inhibited the growth of MDA-MB-231 cells in athymic mice**. The weight of the mice and the tumor volume were recorded every 4 days until animal sacrifice at day 28. **(A) **Tumor volume in each group. **(B) **Tumor weight at the end of the 28-day treatment. **(C) **Photograph of tumors at the end of the treatment. Data presented as mean ± standard deviation (*n *= 6). **P < 0.01 compared with control.

We evaluated the anti-tumor activity of 3,6-DHF in athymic mice xenografted with human tumor models. The tumor volume in 3,6-DHF-treated mice was smaller than that in the control mice on the same measurement day (Figure 2). At the end of the 28-day treatment, the tumor weight in 3,6-DHF-treated mice was less than that in the control mice, and the *T*/*C *values for 10 and 20 mg/kg/day 3,6-DHF treatment groups were 57.2% and 36.4%, respectively. These results indicate that 3,6-DHF could inhibit the growth of breast cancer cells *in vivo*.

### Expression of miR-34a and miR-21 in breast carcinogenesis and the influence of 3,6-dihydroxyflavone

**Figure 3:**
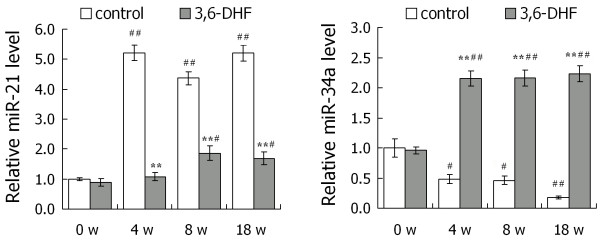
**Expressions of miR-21 and miR-34a in breast carcinogenesis and the influence of 3,6-dihydroxyflavone**. Expression of microRNA-21 (miR-21) and microRNA-34a (miR-34a) in rat breast tissue was detected by quantitative RT-PCR at 0, 4, 8, 18 weeks after 1-methyl-1-nitrosourea injection, respectively. Data presented as mean ± standard deviation (*n *= 3). **P < 0.01 compared with control at each time point. **^#^***P *< 0.05, **^# #^***P *< 0.01 compared with control at 0 weeks.

We detected the expressions of miR-21 and miR-34a in rat breast tissue at 0, 4, 8 and 18 weeks after MNU injection, respectively. The results revealed the global upregulation of miR-21 expression and downregulation of miR-34a expression at all time points in breast carcinogenesis. As shown in Figure 3, 3,6-DHF administration could significantly inhibit the upregulation of miR-21 and effectively increase the expression of miR-34a.

### 3,6-Dihydroxyflavone induced apoptosis of breast cancer cell *in vitro *and *in vivo*

**Figure 4:**
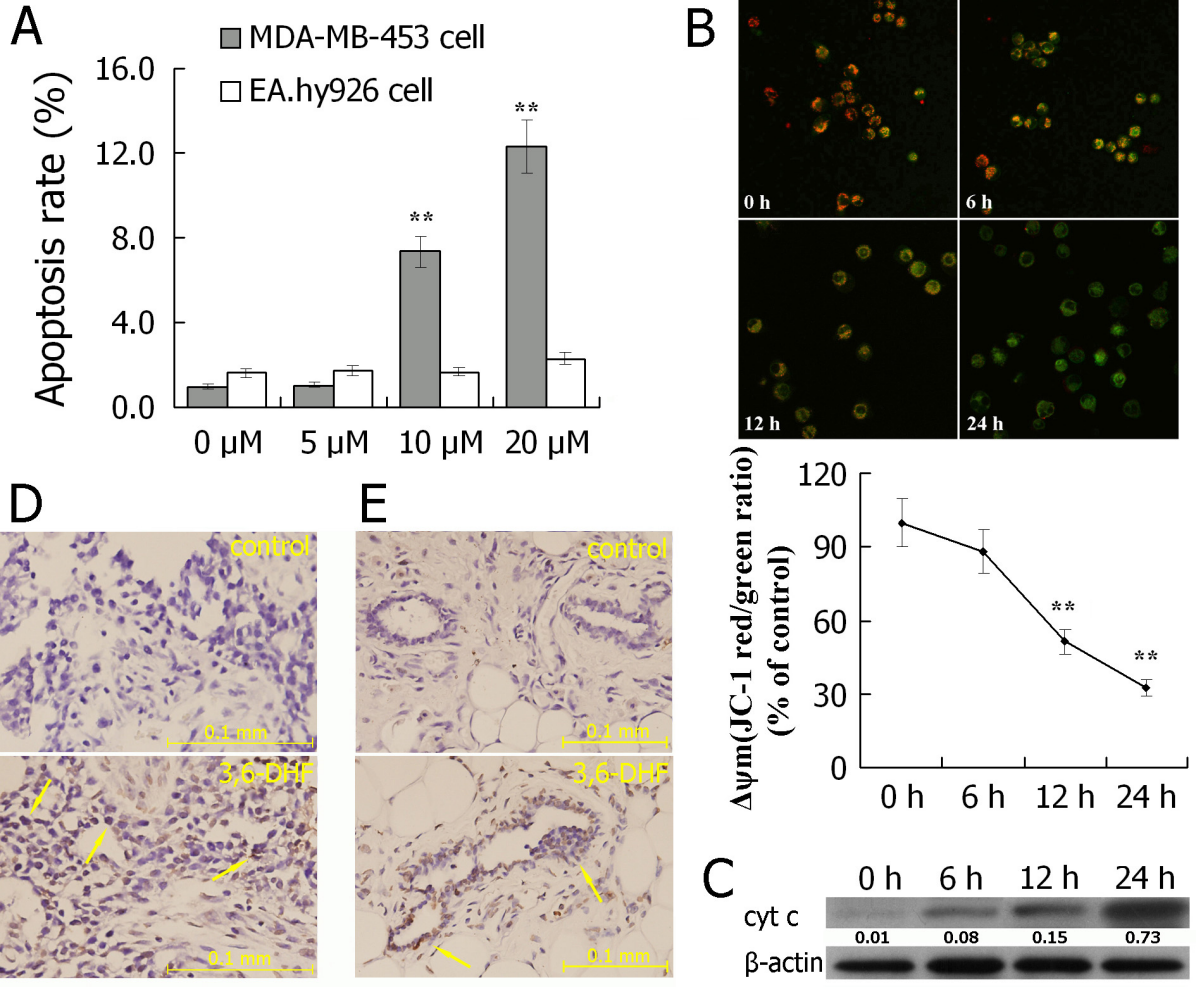
**3,6-Dihydroxyflavone induced apoptosis of breast cancer cells *in vitro *and *in vivo***. **(A) **After being treated with 3,6-dihydroxyflavone (3,6-DHF) for 24 hours at increasing concentrations (5, 10, and 20 μM), apoptosis of breast cancer MDA-MB-453 cells increased in a dose-dependent manner. **(B) **Treatment with 10 μM 3,6-DHF caused the loss of mitochondrial membrane potential in a time-dependent manner. **(C) **Treatment with 10 μM 3,6-DHF induced cytochrome C release in a time-dependent manner. **(D) **Detection of tumor xenograft apoptosis by TUNEL assay. **(E) **Detection of 1-methyl-1-nitrosourea-induced tumor apoptosis by TUNEL assay. Stained nuclei dark brown in color represent apoptotic cells. Arrows indicate apoptotic nuclei. Data presented as mean ± standard deviation (*n *= 3). *P < 0.05, **P < 0.01 compared with control. JC-1, 5,5',6,6'-tetrachloro-1,1',3,3'-tetraethyl-imidacarbocyanine iodide.

Apoptosis analysis using flow cytometry showed that 3,6-DHF induced apoptosis of breast cancer cell in a dose-dependent manner (Figure 4A). We also detected the effect of 3,6-HDF on cell apoptosis in human vascular endothelial cell line EA.hy926, a normal cell line. As shown in Figure 4A, no significant effects were observed. To characterize the upstream factors involved in 3,6-HDF-induced apoptosis in breast cancer cells, the mitochondrial permeability of cells was measured by JC-1 staining-based flow cytometry. As shown in Figure 4B, the significant decrease in the ratio of JC-1 red/green fluorescence indicated a loss of mitochondrial membrane potential. The disruption of mitochondrial membrane was known to result in the release of cytochrome C into the cytosol, which was detected by western blot analysis (Figure 4C). These results revealed that the treatment of breast cancer cells with 3,6-DHF induced apoptosis by decreasing mitochondrial membrane potential and releasing cytochrome C *in vitro*.

We also detected the apoptosis of xenografted tumor and MNU-induced breast tumor by TUNEL assay. As shown in Figure 4D, E, consistent with the results *in vitro*, the immunohistochemical analysis of sections from tumor subjects showed that 3,6-HDF induced apoptosis of breast cancer cells *in vivo*.

### miR-34a and miR-21 play roles in 3,6-dihydroxyflavone-induced apoptosis in breast cancer cells

**Figure 5:**
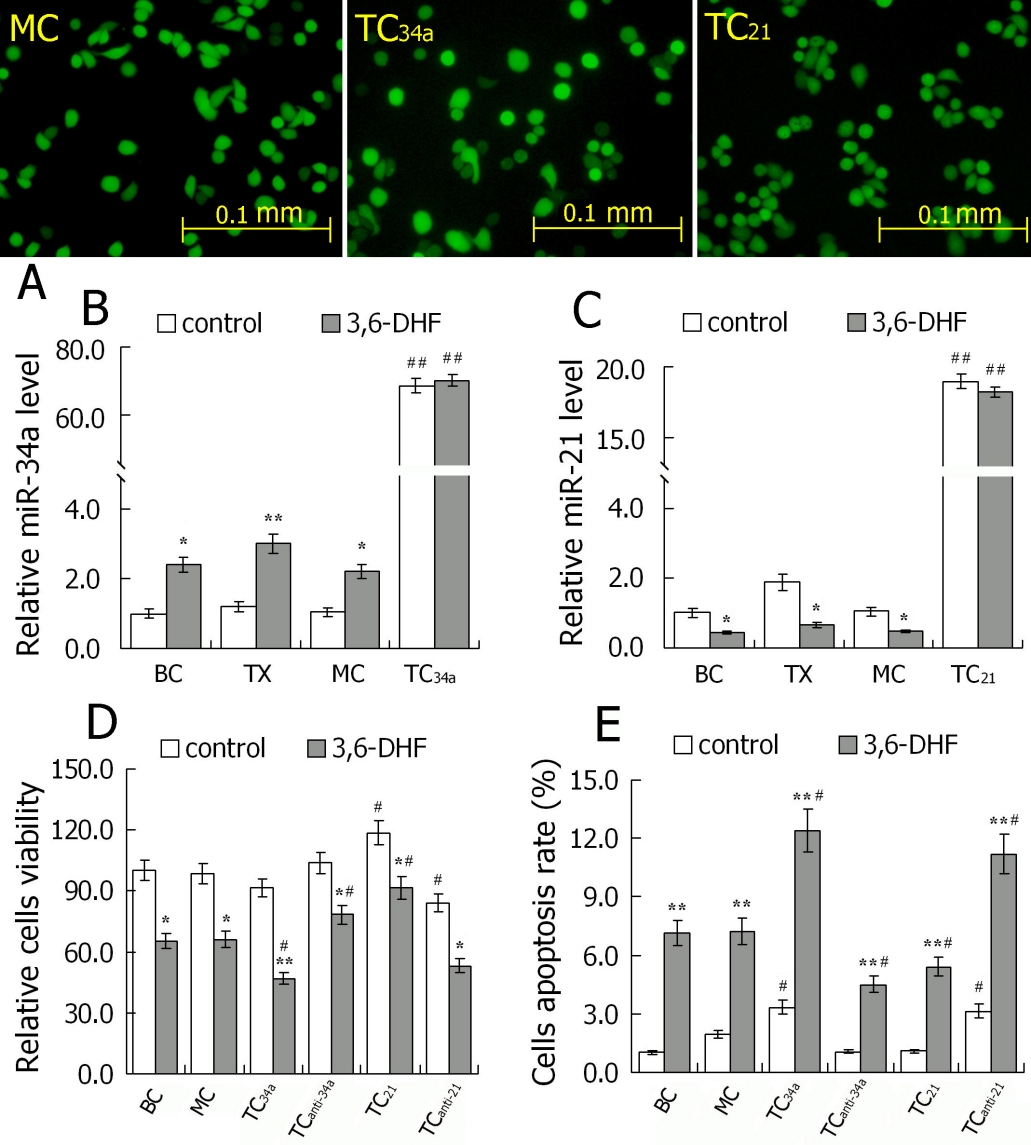
**Increasing intracellular miR-34a or silencing miR-21 expression promoted 3,6-dihydroxyflavone-induced breast cancer cell cytotoxicity and apoptosis**. **(A) **Photomicrographs of transfected cells by fluorescence microscopy. **(B) **Expression of microRNA-34a (miR-34a) in each cell and the effect of 3,6-dihydroxyflavone (3,6-DHF). **(C) **Expression of microRNA-21 (miR-21) in each cell and the effect of 3,6-DHF. **(D) **Effect of 3,6-DHF (10 μM) treatment for 24 hours on cell viability. **(E) **Effect of 3,6-DHF (10 μM) treatment for 24 hours on cell apoptosis. Data presented as mean ± standard deviation (*n *= 3). *P < 0.05, **P < 0.01 compared with control in each group. **^#^***P *< 0.05, **^##^***P *< 0.01 compared with the MDA-MB-453 breast cancer cell (BC) group. TX, tumor xenograft of MDA-MB-231 breast cancer cells; MC, MDA-MB-453 cells transfected with blank plasmids (mock cells); TC_34a_, MDA-MB-453 cells transfected with pcDNA6.2-GW/miR-34a; TC_21_, MDA-MB-453 cells transfected with pcDNA6.2-GW/miR-21. All transfected cells were collected for the next experiments 48 hours after transfection.

Detections using quantitative RT-PCR indicated 3,6-DHF treatment significantly upregulated the miR-34a level and decreased miR-21 expression of breast cancer cells *in vitro *and *in vivo *(Figure 5B, C). To explore the effects of miR-34a and miR-21 in the anticancer activities of 3,6-DHF, we constructed plasmids expressing miR-34a or miR-21, respectively. Transient transfection of these plasmids led to substantial production of mature miR-34a or miR-21 in breast cancer cells (Figure 5B, C). In transfected breast cancer cells, overexpressed miR-34a markedly promoted 3,6-DHF-induced cytotoxicity and apoptosis. Meanwhile, miR-21 overproduction exerted converse affects, compromising the anticancer effects of the flavonoid (Figure 5D, E). Furthermore, a locked nucleic acid oligonucleotide complementary to the miR-34a sequence (anti-miR-34a) served to block miR-34a function. Inhibition of miR-34a in cancer cells led to a marked decrease in 3,6-DHF-induced cytotoxicity and apoptosis. In contrary, inhibition of miR-21 by 2'-*O*-methyl-anti-miR-21 led to a significant increase in 3,6-DHF-induced apoptosis.

## Discussion

Natural compounds and synthetic chemicals that have been shown to inhibit or reverse carcinogenesis have been increasingly adopted in the field of chemoprevention [22,23]. Cancer-preventive phytochemicals have been shown to suppress or block cancer progression by a variety of mechanisms. Development of anticancer agents from flavonoids and other natural products has currently become a very hot topic.

Our previous study identified a flavonoid compound 3,6-DHF as a promising anticancer agent with a very strong cytotoxicity against breast cancer cells and a stronger anticancer activity *in vitro *than apigenin, genistein, quercetin, luteolin, and so forth [16-18]. In the present study, we investigated the anticancer effect of 3,6-DHF *in vivo*. The results indicate that oral administration of 3,6-DHF can effectively suppress MUN-induced mammary carcinogenesis and inhibit the transplanted tumor growth of breast cancer cells *in vivo*. We have so far demonstrated that 3,6-DHF is an effective chemopreventive and chemotherapeutic agent, having efficient anticancer activities against breast cancer cells *in vitro *and *in vivo*. Furthermore, we revealed that 3,6-DHF induced apoptosis of breast cancer cells by decreasing mitochondrial membrane potential and releasing cytochrome C.

miRNAs are well known to play important roles in the pathogenesis of human diseases, including malignancy, and may function as both oncogenes and tumor suppressors - abnormal expression of miRNAs promotes carcinogenesis and cancer progression [24,25]. In this study, we revealed the global upregulation of miR-21 expression and downregulation of miR-34a expression at all the three time points after MNU injection, which could be reversed by 3,6-DHF oral administration. 3,6-DHF significantly upregulated miR-34a expression and decreased miR-21 expression, inducing apoptosis of breast cancer cells *in vitro *and *in vivo*. Furthermore, overexpression of miR-34a induced by plasmid transfection or inhibition of miR-21 by oligonucleotides markedly promoted the pro-apoptotic effect of 3,6-DHF, while inactivation of miR-34a or overproduction of miR-21 compromised the anticancer effects of 3,6-DHF. We hypothesize that the disbalance of cell apoptosis signaling regulation plays an important role in breast tumorigenesis. The flavonoid suppressed carcinogenesis and induced apoptosis of breast cancer cells partially through miR-21 and miR-34a association.

miR-21, a putative oncogene, is most frequently overexpressed in a variety of malignancies, especially in breast cancer, and it has been shown to be implicated in multiple malignancy-related processes including cell proliferation, apoptosis, invasion, and metastasis [26-28]. miR-34a was demonstrated to be a direct transcriptional target of p53, and a component of the p53 transcriptional network [29-31]. miR-34a expression is sufficient to induce apoptosis through p53-dependent mechanisms [32,33]. Loss of function of the p53 tumor suppressor protein causes the pathogenesis of a large fraction of human malignancies [34,35]. Given the importance of pro-apoptosis activity of miR-34a and anti-apoptosis activity of miR-21 in cancer cells, it is interesting to investigate thoroughly their roles in carcinogenesis and anticancer effects of flavonoids.

## Conclusions

Our study shows that oral administration of 3,6-DHF effectively suppresses MNU-induced breast carcinogenesis and transplanted tumor growth *in vivo*, indicating that 3,6-DHF is a potent natural chemopreventive agent. Furthermore, our study also reveals that miR-34a and miR-21 play roles in MNU-induced breast carcinogenesis and the anticancer mechanism of the flavonoid.

## Abbreviations

3,6-DHF: 3,6-dihydroxyflavone; DMEM: Dulbecco's modified Eagle's medium; FITC: fluorescein isothiocyanate; HPLC: high-performance liquid chromatography; JC-1: 5,5',6,6'-tetrachloro-1,1',3,3'-tetraethyl-imidacarbocyanine iodide; miRNA: microRNA; miR-21: microRNA-21; miR-34a: microRNA-34a; MNU: 1-methyl-1-nitrosourea; MTT: 3-(4,5-dimethylthiazol-2-yl)-2,5-diphenyl tetrazolium bromide; PBS: phosphate-buffered saline; PCR: polymerase chain reaction; RT: reverse transcriptase; *T*/*C*: median tumor volume of treated animals/median tumor volume of controlled animals; TUNEL: terminal deoxynucleotidyl transferase-mediated dUTP nick end-labeling; UTR: untranslated region; *V*_t_: tumor volume.

## Competing interests

The authors declare that they have no competing interests.

## Authors' contributions

CH carried out the vector construction and plasmid transfection, and drafted the manuscript. FYJ, YLJ and YL participated in experimental model of carcinogenesis and performed the statistical analysis. XHX and ZY participated in HLPC analysis. ZJD, ZQY and MMT conceived of the study, and participated in its design and coordination and helped to draft the manuscript. All authors read and approved the final manuscript.
